# Medico-Chirurgical Transactions

**Published:** 1847-10

**Authors:** 


### Art. II.

*Medico-Chirurgical Transactions.* Vol. XXIX.—*London,* 1846. 8vo, pp. 352. Eleven Plates.

I. *On the minute anatomy and pathology of Bright's disease of the kidney, and on the relation of the renal disease to those diseases of the liver, heart, and arteries, with which it is commonly associated;* by George Johnson, M.D.

The secreting cells of the kidney, in a healthy state, contain a variable quantity of oil-globules, always considerably less than those of the liver. In kidneys affected with Bright's disease, the quantity of oil-globules is very greatly increased. The true definition, therefore, of the affection is, in Dr. Johnson's opinion, "an exaggeration of the fatty matter which exists naturally in small quantities in the epithelial cells of the healthy organ;" the disease being precisely analogous to the fatty degeneration of the liver. The accumulation does not take place simultaneously and equally in every part of the tubes. Those portions which form the pyramids do not become gorged in any great degree, except in cases where the disease has been of long duration, and in which the cortical portion of the kidney has become wasted. Neither is it common in the expanded portion of the tube which, as Mr. Bowman has shown, forms the investment of the Malpighian plexus.

The cells and tubes, thus engorged, comprise the surrounding capillary plexus, and give rise to congestion of the Malpighian plexus. This congestion leads to transudation of the serum of the blood, and sometimes to rupture of the delicate vessels, and the consequent escape of the colouring matter and fibrin of the blood. These constituents of the blood pass into the tubes, and so become mixed with the urine. Their escape from the blood-vessels is the result therefore of a mechanical impediment to the return of the blood through the veins, precisely as in the experiments performed by Dr. George Robinson, formerly noticed in this Journal. (Brit. and For. Med. Rev. XVIII, 366.) The atrophy of the kidney is also a result of the same compression, which materially interferes with the nutrition of the organ.

It has been frequently observed by pathologists that some affection of the liver coincides with the presence of Bright's disease: this affection Dr. Johnson believes to be most commonly fatty degeneration. In twenty-two cases examined by him, this state of the liver was most marked in 17; and in four of the remaining there was a decided increase of fat in the hepatic cells. He has also observed that in most cases the arteries have been found more or less affected with *atheroma* and *steatoma*, deposits which Mr. Gulliver has shown to be of a fatty nature.

All these facts point in the same direction, and would lead us to look for the source of the disease in some disorder of the processes of digestion and assimilation.

"The processes of primary or secondary assimilation, or both, fail with regard to this fatty matter, which, not undergoing the changes requisite for its ready elimination from the system, or for its application to the nutrition of the tissues, is thrown into the circulation. An effort is made to carry it off by the liver and kidneys; the fat finds its way into the secreting cells of these glands; its escape from these parts in a free state is a slow and uncertain process, and, finding no material in sufficient quantity with which to pass off in a state of combination, the fat accumulates in, and obstructs, the glands."

Such is a sketch of our author's views on this very important subject; they are sufficiently ingenious, but we refrain from giving our assent, until more extended observation shall enable us to decide with more assurance of having found the truth.

II. *Report of a case of ligature of the left subclavian artery, between the scaleni muscles, attended with some peculiar circumstances;* by J. C. Warren, M.D., Professor of Anatomy and Surgery, in Boston, U. S. A.

This is a very remarkable case. The patient, while in a state of intoxication, fell and struck his left shoulder against the kerbstone, on the evening of December 23, 1843. His shoulder was dislocated, and violent efforts were made for its reduction; but as these were supposed to fail, he was sent to the hospital. The parts were so much swollen and bruised, that the condition of the joint could not be ascertained for two days. It was then found that no dislocation existed. On the night of the 20th, he had a violent fit of coughing, and felt something give way in his shoulder. On the 30th, it was discovered that there was no pulse in the left arm, and that sensation and motion were destroyed. There were also some appearances of mortification. On the 27th January, a swelling formed in the axilla, and advanced gradually, till the 4th February, when it burst, and a gush of coagulum, with some fluid dark-coloured blood was discharged. Three days afterwards, there was a sudden and most profuse hemorrhage, which almost proved fatal. He rallied a little during the night, and next morning the subclavian was tied, but with great difficulty. During the operation the pleura was wounded, and a small quantity of air entered the chest. The condition of the patient now gradually improved, and the arm began to assume a more healthy appearance. The ligature separated on the thirteenth day: and seven days afterwards there was some secondary hemorrhage, but it was easily controlled. After this he had two attacks of congestive pneumonia, from which he recovered. Distinct pulsation was not felt in the radial artery until the 4th of February, 1845, 361 days after the operation. On June 15th, his general health was pretty good, and sensation and motion of the arm were slowly improving.

III. *Two cases of disease of the brain, following the application of a ligature to the carotid artery;* by J. P. Vincent, Esq., Surgeon to St. Bartholomew's Hospital.

In the first case, death ensued six days after the operation, and the right hemisphere of the cerebrum was found soft and cream-like. The aneurism was on the right side.

In the second, the right carotid had been wounded by a piece of tobacco-pipe driven through the right side of the root of the tongue. The artery was tied seven days after the accident, on account of profuse bleeding. After the operation, the right side of the body was frequently convulsed, and the left paralysed. This state of matters continued until his death, which occurred on the twelfth day. On dissection, irregular cavities, filled with ash-coloured effusion, and shreds and particles of a greenish hue, were found in the right hemisphere of the cerebrum.

IV. *Case of punctured wound and ligature of the posterior tibial artery, in the upper third of its course;* by James Moncrief Arnott, F.R.S., Surgeon to the Middlesex Hospital.

Experience has shown that the hemorrhage from wounds of this vessel cannot be effectually restrained in all, nor even in a large proportion of cases, by ligature of the femoral artery, and that the general rule holds good that it is advisable, where such a proceeding can be adopted, to secure the vessel at the point of injury. To accomplish this, two methods have been proposed. Most surgeons recommend that the vessel should be reached by incision made on the inner edge of the tibia, between this and the gastrocnemius muscle, which is to be pulled aside, so that the soleus muscle alone is divided. Mr. Guthrie, and some others advise that the incision should be made directly through the whole thickness of the muscles of the calf. The latter was the plan adopted in the case here narrated, and with the most perfect success.

V. *An account of a case in which the corpus callosum, fornix, and septum lucidum were imperfectly formed;* by James Paget, F.R.C.S., Warden of the College, and Lecturer on Physiology at St. Bartholomew's Hospital.

We must refer our readers to the original for a description of the deficiency in this case; without the plate we could scarcely make it intelligible. The patient during life presented no symptoms whatever that would have led to the supposition of any malformation of the brain.

VI. *Case of aneurism, presenting some peculiarities. With remarks;* by Prescott Hewett, Esq., Lecturer on Anatomy at St. George's Hospital School.

In May 1839, Sir B. Brodie tied the external iliac for an aneurism in the groin. The patient, after recovering from a severe attack of peritonitis, was discharged cured. At the latter end of November, slight pulsation returned, but was removed by local pressure applied during two months. In November 1841, there was a slight recurrence of the pulsation, but no increase of size in the tumour. In January 1842, the tumour was larger, but had no pulsation. From this period, it gradually but steadily increased in size for the ensuing twelve months, during which time it grew to the size of the egg of an ostrich; its surface was somewhat irregular, and softer in some parts than in others, although the tumour itself was perfectly solid. During the whole of the time neither pulsation nor sound of any kind could be detected. In January 1843, the tumour became stationary, and some time afterwards it began to diminish; the decrease was continued until July of the same year, when the patient died of phthisis.

On examination after death, the tumour was found lying upon the superficial femoral artery, at about a quarter of an inch below the point where this vessel comes off from the common femoral. It was as large as the head of a full-grown fœtus, slightly irregular on its surface, but perfectly solid. Upon being cut into, it presented the characteristic layers of coagulated blood observed in aneurisms which have been cured. These coagula, which had, for the greater part, lost their colouring matter, were disposed in very thin layers, closely packed together, and completely filling up the aneurismal sac, which was formed by the outer coat of the vessel, and remarkably thin towards its anterior part. The collateral vessels were much enlarged, but there was no abnormal distribution; the return of pulsation, therefore, must be explained by the situation of the tumour, which became affected by the large current of blood brought into its immediate neighbourhood.

VII. *Case of cyanosis of forty years' standing, depending upon congenital obstruction in the pulmonary artery, and patulous foramen ovale;* by Robert S. Spitta, M.B. London, House Surgeon to St. George's Hospital.

VIII. *On the ganglionic character of the arachnoid membrane of the brain and spinal marrow;* by George Rainey, M.R.C.S.

Mr. Rainey conceived the first idea of the resemblance between the arachnoid and sympathetic, from observing at the junction of two of the chords situated between the arachnoid and pia mater, a triangular body of the form and general appearance of a ganglion. He also observed a branch going from the chord connected with this body to the arachnoid membrane, along which it ran for a considerable distance, dividing and subdividing in its course, in the manner of a nerve; the successive subdivisions becoming more and more minute, and at the same time interlacing and inclosing areolæ filled with corpuscular matter. These corpuscles were so blended with the ultimate filaments of this chord, as to render indistinct their exact mode of termination. On further examination, he found that the opposite extremity of such a chord terminated by ramifying either on an artery or on a cerebro-spinal nerve.

The disposition of the ramifying filaments of the arachnoidal chords, and the form and size of the gangliform plexuses connected with them, bear some proportion to the number and size of the vessels in their vicinity. Hence, about the base of the brain, where the branches of the arteries are large, the plexuses are also large, and of an irregular shape, whilst on its upper surface, where the vessels are comparatively small, and more equal in size, and have a more uniform distribution, the plexuses also are smaller, more numerous, and more regular in their shape and size.

Besides the plexuses situated in the course of the chords of the arachnoid, there are others which are more intimately connected with its cerebral surface, and which, in some situations, appear to compose the entire thickness of the membrane. In these plexuses, the filaments interlace very much in the same manner as the nerves do in the plexuses of the cerebro-spinal and sympathetic systems.

On comparing the filaments composing the arachnoidal chords with filaments of undoubted sympathetic nerves, Mr. Rainey found a very close resemblance. The nuclear fibre, described by Henle, was very rare; but the other two forms which exist in the sympathetic were exceedingly numerous, viz. that which consists of bundles of minute wavy filaments, intermixed with small particles of granular matter; and that which is made up of similar filaments, not always united in bundles, but apparently interwoven, and often totally destitute of granular matter.

Some of the plexuses on the cerebral surface of the arachnoid have the interstices, formed by their interlacing fibres, completely filled up with small roundish corpuscles, about the size of blood-discs; whilst, in others, these fibres are covered with irregularly oval masses of them. On this surface also, in various situations, there are well-defined round or oval bodies, having in their centre a granular nucleus surrounded by fibrous tissue, intermixed with more or less corpuscular matter. Of the nature and office of these bodies Mr. Rainey professes himself ignorant.

It is only those portions of the membrane which are made up of soft corpuscular matter, and the ramifications of the chords going to the arteries and nerves, which the author considers to be of a nervous character. We feel that much more extended observation is required before general assent can be given to views so novel. Two plates are given in illustration.

IX. *An account of a case of partial double monstrosity.* (*Ischiopage symelien of Geoffroy Saint Hilaire, Heteradelphia of Vrolik*)*;* by W. Acton, Esq., Surgeon to the Islington Dispensary.

The subject was a healthy male Portuguese child, six months old. We quote Mr. Acton's description.

"The child is exhibited lying on its back in a little cot; is lively and good-looking, and well-proportioned, both in the upper and lower extremities, the peculiarities being confined to the parts below the umbilicus; a truss is worn on account of an umbilical hernia. Below the umbilicus, and to the right and left of the mesial line, are two distinct penes, each as large as the penis of a child of six months old: their direction is normal. I may mention that water passed from both organs at the same moment during the time that Dr. Cursham and Mr. Perry were examining the infant with me. Each penis is provided with a scrotum, the outer half of each scrotum containing one testicle, the inner half of the scrotum is far removed from the outer; and the two inner halves appear like another scrotum between the two penes. Between and behind the legs of the child we see another limb, or rather two lower extremities united together in their whole length. The upper part of this compound limb is connected to the rami of the pubis by a short stem, half an inch in length, and as large as the little finger, apparently consisting of separate bones or cartilage, for, on moving the compound limb, at the same moment the finger is kept on the stem, crepitation is felt, but I could not detect any pulsation. Immediately beyond this stem, and concealing it, the compound limb assumes a size as large as the combined natural thighs of the child, and within the upper part irregular portions of bone may be felt (probably a portion of a pelvis and the heads of the thigh-bones), which may be traced down, united together into one mass, to a leg of comparative small size, though still larger than either of the healthy legs, and terminated by a double foot in the position of *talipes*, with the sole turned forwards, and furnished with ten toes, the two great toes being in the centre of the others: the two outer toes on each side are webbed.

"When the child is placed on its belly, the spine and back present a perfectly normal appearance; the anus is in its usual situation; the functions of the bowels are duly performed. Viewed in this position the compound limb assumes a roundness and fulness equal to the buttocks of a young child, and a slight depression is observed, as if for the anus. Tracing the limb downwards, we find only one patella, which is on the same aspect of the limb as the anus; the joint bends freely, and the compound extremity terminates as above described. This compound limb is quite motionless, the upper portion alone appears endowed with sensibility; its vitality seems low, as the toes have a blueish appearance. The upper portion, however, is of the same temperature as the body of the child."

X. *Some remarks on wounded arteries, secondary hemorrhage, and false aneurisms;* by Robert Liston, F.R.S., Surgeon to the University College Hospital, and Vice-President of the Society.

Mr. Liston's valuable practical remarks have relation to the well-known case of Mr. S. who was wounded in a duel at Gosport, in May 1845. The ball traversed the course of the femoral vessels a little above, and in front of the trochanter major. There was great immediate hemorrhage, and this was succeeded by swelling and all the symptoms of false aneurism.

"The nature of the case was very apparent. A large false aneurism, not well bounded, and rapidly increasing over the abdominal parietes in the iliac region, and arising from a wound of the femoral artery, or of some branch, divided close to its origin, had to be arrested in its progress, otherwise the patient must be left exposed to the risk of perishing suddenly, and at no distant period.

"After consultations on the evening of the 30th and morning of the 31st, with Drs. Mortimer and Stewart, Messrs. Jenkins, Sampson, and Potter, the external iliac artery was tied, with the loss of not more than a table-spoonful of blood, and with the immediate effect of arresting the pulsation, and of removing in a great measure the tension of the tumour. The corresponding limb, which had been somewhat swollen, was found, on the day after the operation, diminished in bulk, and of its natural temperature.

"Symptoms of peritonitis supervened this same evening, and on the following afternoon the patient sunk." (pp. 108-9.)

On examination after death, it was found that the injured artery was one of the superficial branches of the femoral artery, and was divided about half an inch below Poupart's ligament, and about an inch from the main body of the artery. The following summary explains and amply justifies all proceedings had recourse to in the case.

"I have thus endeavoured to show—

"1st. That the case of Mr. S. was one of great and immediate danger.

"2dly. That some decisive step required to be taken, and that without a day's delay, in order to avert the danger that impended over him.

"3dly. That very great risk would have been incurred in searching for and attempting to put a ligature upon the wounded part of the vessel.

"4thly. That there is ample authority for adopting the step which was had recourse to in the case. Ligature of the external iliac was undertaken, in order to ward off the expected fatal termination, through a sudden escape of arterial blood.

"And it may here be remarked, that we were not apprehensive of a remote danger, or labouring under a vague impression that something untoward might arise; the danger was manifest. An artery had begun to pour out its contents impetuously, and the blood was only prevented from escaping externally by rotten tissues, that were about to separate, and could not by any possibility resist the impulse many hours longer. It must also be borne in mind, in considering the bearings of this case, that it might have been absolutely impossible to effect even the operation of tying the iliac, had the effusion extended ever so little further. No one will pretend to say that the operation of tying the iliac artery of itself is not attended with danger to life. But the dangers of mortification, secondary bleeding, &c., likely to arise from the operation, were considered as weighing but lightly in the scale against those impending from the effects of the pistol-shot. Mr. S. died, it has been seen, from the effects of inflammation of the peritoneal cavity. But this was scarcely taken into account at all, in consulting on the case. When we look at the result of the cases published in this country, from the time that Mr. Abernethy first tied the vessel, about fifty years ago, to the present period, it will be seen that we were justified in not laying very great stress on this source of danger. It appears that out of 45 cases recorded, (and collected by Mr. Crisp, in an excellent paper on aneurism, to which was lately awarded the Jacksonian prize by the Council of the College of Surgeons,) 9, or one fifth, died; three of those from sloughing and bursting of the sac, two from mortification of the limb, two from secondary hemorrhage, one from disease of the chest, and one from sinking of the vital powers. Many unsuccessful cases have no doubt been left unpublished, and some in which the patients suffered from inflammation of the peritoneal membrane might be adduced.

"In the case of Mr. S., the sac no doubt might have sloughed, secondary bleeding might have occurred on the separation of the ligature, however carefully applied. But there was nothing to prevent the collateral circulation from being carried on freely, and nothing connected with the wound, or consequent aneurismal swelling, to prevent the free return of blood by the veins. In the opinion of those concerned, the step most likely to avert danger, and prolong the patient's life, was adopted; the only step, it has been shown, that could have possibly been resorted to with propriety or safety." (pp. 128-30.)

XI. *Case in which a large tumour was developed in the substance of the fifth nerve and its ganglion;* by James Dixon, Esq., Surgeon to the Royal London Ophthalmic Hospital.

XII. *On the capacity of the lungs, and on the respiratory functions, with a view of establishing a precise and easy method of detecting disease by the spirometer;* by John Hutchinson, Surgeon.

It is quite impossible to give anything like a full analysis of this most elaborate production, but we have no hesitation in recording our deliberate opinion, that it forms one of the most valuable contributions to physiological science that we have met with for some time. In all future investigations into the phenomena of the respiratory processes, the name of Mr. Hutchinson must receive honorable notice. His inquiries have been made upon a very large scale, 2130 individuals of various ages and employments, of both sexes, in health and in disease, having been subjected to examination.

The quantity of air which may be contained in the chest, under different circumstances, is divided by Mr. Hutchinson into four portions. 1. *Breathing air*, or that which is drawn in and expelled during ordinary respiration. 2. *Complemental air*, or that which may be introduced by the deepest possible inspiration. 3. *Reserve air*, or that portion which may be expelled by the fullest expiration. 4. *Residual air*, or that portion which always remains in the lungs, and over which we have no control. The first three, taken together, form what he denominates the *vital capacity* of the chest, and it is this which is measured by the spirometer.

Now this vital capacity is found to be modified in a determinate manner by certain conditions, viz. the height, weight, and age of the individual, supposed to be healthy. Of these, by far the most important is height, *why* we cannot tell, but the number of observations is too great, and the results derived are too uniform to admit of the least doubt. It appears, then, that for every inch of height (from 5ft. to 6ft.) eight additional cubic inches of air, at 60°, are given out by a forced expiration.

Weight has a less decided influence. At the height of 5½ feet, the vital capacity is not affected by any variations of weight under 161 lbs. or 11½ stone; but above this point it is diminished in the relation of 1 cubic inch for every lb. up to 196 lbs., or 14 stone. At other heights, between 5ft. 1 inch, and 5ft. 11 inches, ten per cent. may be added to the mean height, before we allow the weight to affect the vital capacity in the relation of one cubic inch per lb.

In regard to age, from 35 to 65, there is a decrease of rather more than one cubic inch per year.

We subjoin a very useful table showing the *normal* vital capacity in relation to height. The third column gives the result of arithmetical calculation.

| Height. | | From Observation. | Regular Progression. | Height. | | From Observation. | Regular Progression |
|---|---|---|---|---|---|---|---|
| ft. in. | ft. in. | cub. in. | cub. in. | ft. in. | ft. in. | cub. in. | cub. in. |
| 5 0 to | 5 1 | 174 | 174 | 5 6 to | 5 7 | 229 | 222 |
| 5 1 | 5 2 | 177 | 182 | 5 7 | 5 8 | 228 | 230 |
| 5 2 | 5 3 | 189 | 190 | 5 8 | 5 9 | 237 | 238 |
| 5 3 | 5 4 | 193 | 198 | 5 9 | 5 10 | 246 | 246 |
| 5 4 | 5 5 | 201 | 206 | 5 10 | 5 11 | 247 | 254 |
| 5 5 | 5 6 | 214 | 214 | 5 11 | 6 0 | 259 | 262 |

In prosecuting his inquiries, Mr. Hutchinson has arrived at some results, which are totally at variance with generally received opinions. Thus, he does not find that width or depth of chest exercise any special influence over the vital capacity. Tall persons with narrow chests invariably expire more than shorter individuals with apparently more capacious chests. It appears that the mobility of the walls of the thorax has very considerable influence.

He has also shown that the ordinary descriptions of the movements of the chest and abdomen are erroneous, but for information on this, and many other points of great interest, such as the elastic power of the ribs, the action of the intercostal muscles, &c., we must refer the reader to the original; assuring him that by a careful perusal he will not fail to gain much new and useful knowledge.

XIII. *Hydatid cyst, either originating in, or pressing upon, the prostate gland;* by George Lowdell, late House Surgeon to the Sussex County Hospital.

XIV. *Cases of varicocele, treated by pressure, with observations;* by T. B. Curling, Lecturer on Surgery, and Assistant Surgeon at the London Hospital.

Mr. Curling relates several cases in which a cure was effected, and some in which great relief was obtained by the application of a moc-main lever truss, at the external abdominal ring.

XV. *Account of a case of congenital deficiency of one kidney, with granular degeneration of the existing one;* by G. Busk, Surgeon to the Seaman's Hospital Ship "Dreadnought."

This is an interesting case. The subject, a gentleman æt. 27, had enjoyed good health until within the last three years of his life. At this period he became bloated and pale, and incapable of as much exertion as formerly. He did not come under medical treatment, until about $2\frac{1}{2}$ months before his death; but previously to this he had taken a considerable quantity of calomel, by the advice of a friend, and was salivated at the time when he was first seen by Mr. Ceeley, of Aylesbury. The urine was albuminous. He was very subject to epistaxis. When the ptyalism subsided, a small crack-like sore was observed on the frænum linguæ, from which blood constantly oozed. This never healed, and a few days before death the under side of the tongue and the inner surface of the cheeks became gangrenous.

On examination after death, the heart was found hypertrophied to about double its natural size. All the valves were healthy. The liver was about one third larger than usual. It did not grease the scalpel, but under the microscope, presented a large quantity of oil. There was no trace whatever of the left kidney or supra-renal capsule. The right kidney was about 2 inches in length, by $1\frac{1}{4}$ in width, and presented externally a contracted or corrugated aspect. It was very pale, speckled with white, and on a section, scarcely any distinction of substances was observable. The whole presented a dense cicatriform aspect, of a semi-transparent hue, sprinkled with small opaque specks, and, in the remains of the tubular portions, marked with white striæ, obviously vessels filled with a dense white material. The pelvis was remarkably small, and the maxillary processes appeared also to be unusually few in number.

Under the microscope, the tubuli uriniferi were found to be in parts indistinct or obliterated, and in others to be filled with a semi-opaque, white, granular material, soluble, or rendered transparent by acetic acid, and presenting none of the characters of oil, very few globules of which were found in any part of the gland.

This case evidently does not bear out Dr. Johnson's views of the pathology of Bright's disease.

XVI. *On a particular derangement of the structure of the spleen;* by J. B. S. Jackson, M.D., Boston, U. S. A. Communicated (with some introductory remarks and comments) by Thomas Hodgkin, M.D.

This pathological change was described by Dr. Hodgkin in 1832, and by him ascribed to the extravasation of blood caused by local injury. Professor Rokitansky, on inspecting his specimen, recognised the appearance as one familiar to him, but affirmed his belief that it was one of the effects of endocarditis, and was produced by the poisoned condition of the blood. Recently, Dr. Hodgkin has received a letter from Dr. Jackson, of Boston, recording his experience. Part of this we shall quote, and our readers will learn from it, both what the morbid appearance in question is, and what its connexion with cardiac disease.

"The whole number of cases is nine. The disease is found in regular masses, varying from a quarter of an inch to one inch in diameter; and generally several were found in the same organ. These were situated in the edge, or very near to it, and not upon the two broad faces of the organ; and they appeared to extend from the surface into the substance of the organ, being never, I believe, found in the substance without coming to the surface. They were perfectly defined, and often surrounded by a little loose cellular tissue, but were never encysted. The colour was the striking characteristic; it was always more or less yellow, except in one case, where it was evidently stained with blood; sometimes a faint yellow, but more generally bright. In one case it is described as an 'ochre;' in the second, as a 'gamboge' (i. e. gamboge when moistened by water); and in a third, as being of a bright golden yellow colour, contrasting strongly and very beautifully with the deep red colour of the rest of the organ. Sometimes in the less perfected specimens there was an intermixture of red. The masses are perfectly opaque, the cut surface is smooth, and generally they are of the consistence of fibrin; blood-vessels were distinct in some specimens, but generally they could not be seen. The surface of the organ corresponding to these masses was opaque, the yellow colour showing through, more or less from beneath; but in one case, the disease was only marked externally by a deep red colour; the whole surface was sometimes depressed, and sometimes not at all so, but more frequently there was a narrow depression around the limits of the disease; old peritoneal adhesions were sometimes found in this part. The spleen itself was generally noted as of the usual size.........Nothing was ever seen in the organ like tubercular or malignant disease, nor was there any trace of pus either in or out of the diseased masses.........You are inclined to regard it as the effect of external injury.... but, in my cases, no allusion is made to any such explanation, the fact being overlooked, if it existed, as I was not aware at the time of your observations. In five of the cases it is expressly stated that there were no tubercles in the lungs—an interesting point, as bearing of the nature of the disease of the spleen; in two, who died of anything but pulmonary complaint, the fact is not noticed; in one there existed a few latent tubercles, and one patient only died of phthisis. ........One thing, however, was common to every one of the nine, whatever was the disease that caused death,—and that was, an organic disease of the heart; and, in five of the nine, there were found vegetations upon the valves; in one of these last there was fibrin adhering to the inner surface of the left auricle, and becoming organized; in another (not one of the five) there was, in the appendix of the right auricle, a mass of fibrin as large as the top of the thumb, and completely infiltrated with pus, as some would say; in one of the five cases of vegetations of the valves there was also arteritis."

XVII. *On a luminous appearance of the human eye, and its application to the detection of disease of the retina and posterior part of the eye;* by William Cumming, late House Surgeon to the London Hospital.

Mr. Cumming has observed that the healthy human eye, when properly examined, presents a luminous appearance, like that of cats, dogs, &c. To detect this, it is necessary that the eye should be at some distance from the source of light; the distance being greater in proportion to the intensity; that the rays of light diffused around the patient (and sometimes around the eye itself) should be excluded; and that the observer should occupy a position as near as possible to the direct line between the source of light and the eye examined. These conditions may generally be obtained by placing the person to be examined in a dark room, and looking at his eye through a half-opened door.

In a few cases of disease of the retina he has found this luminousness destroyed or lessened, and he proposes it as a means of diagnosis.

XVIII. *Account of a case in which an abscess in the neck communicated*

*by an ulcerated opening with the arch of the aorta, and in which the hemorrhage did not prove fatal in less than forty-eight hours ;* by George Busk, Surgeon to the Hospital Ship, "Dreadnought."

XIX. *On the intimate structure of the human kidney, and on the changes which its several parts undergo in "Bright's Disease ;"* by Joseph Toynbee, F.R.S., one of the surgeons to the St. George's and St. James's General Dispensary.

This valuable and interesting paper details the results of a series of investigations undertaken by Dr. Bright and Mr. Toynbee. Without the aid of plates, it is scarcely possible to convey an accurate idea of parts, so minute and so complicated. We shall, however, attempt at least to bring before our readers so much as will enable them to understand the pathological views of our author; and in doing this we shall presume them to have a general notion of the minute anatomy of the organ, and shall not, therefore, occupy either their time or our own by explanations, which to one unacquainted with the subject would be absolutely necessary.

The *parenchyma* of the kidney consists of active cells and corpuscles, which recent investigations lead us to believe bear an important part in the processes of development, nutrition, and secretion. It is probable that the blood is by them, in some measure, prepared for that further change which takes place during its diminution, by the agency of the epithelial cells, in its passage into the interior of the excretory duct. Mr. Toynbee is of opinion, from the results of frequent examinations, that the nervous filaments end by becoming continuous with the parenchyma: a fact, if established, of great importance.

Near the apices of the mamillary processes, the *tubuli uriniferi* are closely packed together; at the bases they expand, and in the cortical substance they are dispersed in all directions, and in a complicated manner. They are more regularly arranged on the surface of the kidney, to which, when successfully injected, they give a lobular aspect. Mr. Toynbee is at variance with Mr. Bowman as to the mode of termination of the tubuli, the latter denying that they ever communicate with each other. The following is Mr. Toynbee's view of the true relation of the tubuli to the *corpora malpighiana* :

"1st. The capsule of the corpus malpighianum, instead of being, as supposed, an expansion of the tubule, is a distinct globular investment, enveloping both the tubule and the tuft of vessels. 2d. This globular investment is neither continuous with the tubule, nor with the blood-vessels, but is expanded over them. 3d. Into one part of the capsule the artery enters, while the other receives the tubule. 4th. The artery divides and subdivides, so as to form a globular mass of capillaries in the interior of the capsule from which the efferent vessel emerges. 5th. The tubule, after penetrating the capsule, becomes tortuous, and twists into a coil, and after being in contact with the ramifications of the corpus, it emerges from the capsule."

The capsule of the corpus malpighianum is a thin transparent membrane, without fibres or cells. Its contents consist of convolutions of vessels closely aggregated together, and apparently attached to a central vessel from which they radiate.

The venous trunks of the kidney commence on the surface of the organ, in small stellated branches, which passing through the cortex, are there considerably increased in size by the junction of the numerous ramifications at the bases of the pyramidal masses. The veins situated in the tubular region having entered them, they, by their union, form the larger vessels which surround the pelvis, and terminate at length in the renal vein.

*Pathological observations.* Mr. Toynbee adopts Dr. Johnson's views of the nature of Bright's disease, but differs from him in believing that congestion is the first step in the process. He recognises three stages of the disease. In the *first,* the kidney is enlarged, and innumerable black points are visible, which are the dilated corpora malpighiana seen through the capsule. The white spots, which derive their appearance from the collection of fatty matter, begin to be perceptible. The peculiar features of this stage consist of an enlargement of the arteries entering the corpora malpighiana; the dilatation of the vessels of the tuft, the capillaries and the veins; an increase in the size of the capsule of the corpus and of the tubuli, and a large addition to the quantity of the parenchyma of the organ.

In the *second* stage, the organ is very greatly increased in size, and its tissue flabby; the surface is smooth, and presents numerous white spots; and the capsule is but slightly adherent. The artery of the corpus malpighianum is so greatly enlarged, that frequently it equals the dimensions of the tube itself, and is eight or ten times its natural size. The vessels forming the tuft are likewise enormously enlarged. (This is the very opposite of Mr. Bowman's opinion.) The tubuli, also, are increased in size, aggregated in masses, and extremely convoluted.

In the *third* stage, the kidneys are smaller than natural, hard, white granules are prominent on their surface, which is more or less lobulated, and marked by the stellated arrangement of the veins; the capsule is adherent; vesicles of large size are frequently everywhere interspersed; and numbers of smaller ones stud the whole surface. The arteries are more contracted than in the second stage, and the malpighian tuft is so often changed from its natural state, that the greater part of its vessels are not capable of being injected. The capsule of the corpus has assumed a more contracted appearance. The tubuli are larger than in the preceding stage, and are gathered into rounded masses, which form the granules on the surface of the organ. The latter are of a white hue, and are most commonly fully distended with fatty depositions; though not unfrequently they appear like dark spots, the tubuli being full of blood. The parenchyma is hard, and is composed of elongated stellated cells, from the angles of which fine threads proceed, and communicate with each other.

The paper is illustrated by five beautiful plates.

XX. *On the relation between the constituents of the food and the systems of animals;* by Robert Dundas Thomson, M.D., Lecturer on Practical Chemistry in the University of Glasgow.

This fragment constitutes another step in the right direction. We sincerely trust the author will pursue his investigations, which cannot fail at length to lead to valuable results.

